# Work-Related Stress and Behavioural Correlates of Lower Urinary Tract Symptom Profiles in Female Nurses: A Latent Class Analysis Based on the Nurse Urinary Related Health Study: Four Profiles of Lower Urinary Tract Symptoms in Female Nurses

**DOI:** 10.1155/2024/7318901

**Published:** 2024-10-17

**Authors:** Jie Gao, Dongjuan Xu, Chen Wu, Ming Li, Jieqiong Ren, Yongjuan Rao, Kefang Wang

**Affiliations:** ^1^School of Nursing and Rehabilitation, Shandong University, Jinan, Shandong Province, China; ^2^School of Nursing, Purdue University, West Lafayette, Indiana, USA

**Keywords:** female nurses, latent class analysis, lower urinary tract symptoms, voiding behaviors, work environment

## Abstract

**Aims:** This study aimed to identify symptom clusters in a population-based sample of female nurses who reported experiencing lower urinary tract symptoms (LUTS) and examine distinct risk factor profiles within each symptom cluster, with an emphasis on modifiable lifestyle and work-related factors.

**Materials and Methods:** This study included 6735 female nurses who reported experiencing at least one LUTS. Latent class analysis was conducted to identify symptom clusters by jointly modelling 11 symptoms. Analysis of variance, chi-square tests and multinomial logistic regression analyses were conducted to examine distinct risk factor profiles within each symptom cluster.

**Results:** On average, female nurses experienced 2.2 symptoms. Four clusters were identified: the multiple severe symptoms (*n* = 546, 8%), incontinence symptoms (*n* = 2089, 31%), urgency–hesitancy symptoms (*n* = 3357, 50%) and nocturia symptoms (*n* = 743, 11%). Unique profiles of female nurses were associated with each symptom cluster and demonstrated the influential role of work-related factors in the development of LUTS. These factors included a demanding workload, heightened levels of perceived stress, extended work hours, engagement in night shifts, infrequent and delayed voiding behaviours and restriction of fluid intake.

**Conclusions:** Our findings substantiate the vulnerability of female nurses to LUTS, which is attributable to their demanding work environments. LUTS among female nurses should be recognised as an occupational hazard and remains an understood topic in the workplace, warranting attention and tailoring of intervention strategies.

**Implications for Nursing Management:** Nurse managers should be aware that LUTS represent an occupational hazard for nurses and that high-stress work environments and inappropriate urination behaviours adversely affect nurses' bladder health. Addressing LUTS-related issues requires increased staffing levels along with a fundamental shift in workplace culture. Fostering a culture of self-care with an emphasis on bladder health promotion is imperative for nursing professionals and employed women.

## 1. Introduction

Lower urinary tract symptoms (LUTS) are highly prevalent among women, with studies estimating 12.6%–89.6% of women report at least one LUTS [1, 2]. These symptoms significantly interfere with daily activities and impose an economic burden on both individuals and society [3, 4]. According to the International Continence Society (ICS) criteria, LUTS includes a wide range of storage, voiding and postvoiding symptoms [5]. Clinical research and diagnosis have mainly focused on these categories, or predefined conditions like overactive bladder (OAB), or a single core symptom such as nocturia or stress urinary incontinence (SUI) [6]. However, individuals often exhibit complex symptom combinations, such as urinary incontinence (UI) alongside voiding or storage symptoms [7], and nocturia combined with frequency [8]. Given the clinical heterogeneity of LUTS, the underlying mechanistic pathways can plausibly vary across distinct patient subgroups. Therefore, identifying various LUTS patterns or clusters could improve precise prevention and management strategies.

Three studies investigated the clustering of specific symptoms in women with LUTS. One study predominately involved white women from the UK, Sweden, Italy, Germany and Canada (the EPIC survey) [9]; another included community-dwelling women who lived in Boston, MA, US (the Boston Area Community Health survey: BACH survey) [8]; and a third focused on care-seeking women from six US tertiary care centres (the Symptoms of Lower Urinary Tract Dysfunction Research Network: LURN study) [6]. Women with LUTS were classified into four clusters in the BACH and LURN studies and into six clusters in the EPIC survey. However, no studies have examined LUTS clusters among female nurses despite their heightened vulnerability and higher prevalence of LUTS compared to the general female population [2, 10, 11].

Several factors have been found to be associated with LUTS, including demographic factors such as age, health-related factors (e.g., obesity and chronic conditions) and lifestyle factors (e.g., fluid intake) [12, 13]. For female nurses, work-related factors such as occupational stress and heavy workloads contribute significantly to the development or exacerbation of LUTS [12]. These factors are particularly noteworthy in China, where the nurse-to-patient ratio is significantly lower than that in developed countries [14, 15]. Notably, most studies investigating LUTS clusters failed to explore distinct risk factor profiles within each identified symptom cluster. A comprehensive understanding of these symptom clusters and their corresponding risk factor profiles can both enhance our insight into the potential shared aetiologies of various LUTS and facilitate targeted interventions, thereby optimising treatment outcomes. Thus, this study aimed to identify symptom clusters in a population-based sample of female nurses in China who reported experiencing LUTS and examined distinct profiles of risk factors within each symptom cluster, with a particular emphasis on modifiable lifestyle and work-related factors.

## 2. Materials and Methods

### 2.1. Data Source, Sample and Data Collection

This study used baseline data from the Nurse Urinary Related Health Study (NURS) that was conducted between November 2020 and February 2021. The NURS is an online, population-based, prospective longitudinal study that surveys approximately 15,000 female nurses every two years. The details of the survey design, sampling frame and selection are described elsewhere [16]. A two-stage clustered sampling technique was used to select female nurses from hospitals in Shandong Province, China. The sampling process involved stratifying the province into four economic groups based on gross domestic product (GDP) and subsequently categorising hospitals within these regions into two levels (Levels A and B). Twenty percentage of hospitals in each stratum were randomly selected. All registered female nurses from the selected hospitals were invited to participate voluntarily.

At baseline, 17,999 female nurses from 20 hospitals were invited to participate. Among them, 2829 (15.7%) did not respond, and 207 (1.2%) refused to participate. A total of 14,900 female nurses participated in the NURS. The inclusion criterion for this study was reporting the experience of LUTS measured by the International Consultation on Incontinence Questionnaire-Female Lower Urinary Tract Symptoms (ICIQ-FLUTS). A detailed definition of LUTS is provided in the Measures section. Exclusion criteria included the following: (1) pregnancy; (2) a urinary tract infection in the previous month or three times per year; (3) lower urinary tract injury or disease; (4) pelvic or genitourinary surgery within the past 3 months; (5) chemotherapy or other treatments for cancer; (6) spinal cord injury, cerebrovascular diseases or nephropathy; and (7) the use of diuretics. The final study sample included 6735 female nurses. The study was approved by the Institutional Review Board of the University and all selected hospitals.

### 2.2. Measures

#### 2.2.1. LUTS

The validated Chinese version of the ICIQ-FLUTS was used to assess 11 LUTS (i.e., nocturia, frequency, urgency, bladder pain, hesitancy, straining, intermittency, urge urinary incontinence (UUI), SUI, unexplained UI and nocturnal enuresis) [17]. Nocturia was defined as the need to urinate at least twice a night [15]. Frequency was defined as urination more than eight times a day. The remaining nine symptoms were rated on a five-point scale (0 = *never*, 1 = *occasionally*, 2 = *sometimes*, 3 = *most of the time* and 4 = *all of the time*). Participants who reported sometimes/most of the time or all of the time were considered symptomatic. Participants were considered to have LUTS if they experienced at least one of the 11 symptoms. The ICIQ-FLUTS has been validated with Chinese female nurses with good reliability and validity [17]. Cronbach's alpha coefficient of the scale in this study was 0.79.

#### 2.2.2. Work-Related Variables

Years of practice (≤ 5, 5–10 and > 10), weekly working hours (≤ 40 and > 40), working night shifts, hospital level (Level A or Level B) and perceived stress were reported by participants. Level A hospitals typically have more hospital beds, improved quality of care, advanced treatments and technologies, and higher patient acuity and workloads than Level B hospitals. The four-item Perceived Stress Scale was used to assess perceived stress, with higher scores indicating greater stress [18]. In this study, Cronbach's alpha coefficient for perceived stress was 0.61.

#### 2.2.3. Lifestyle Variables

Coffee and tea consumption, fluid intake levels (≤ 500 mL, 501–1500 mL and > 1500 mL) and frequency of delayed voiding (never, rarely, sometimes, often and always) were assessed. Participants who reported that they sometimes/often/always delayed voiding were categorised as those adopting delayed voiding behaviour.

#### 2.2.4. Health-Related Variables

Obesity (body mass index ≥ 28 kg/m^2^), parity (nulliparous and ≥ 1 childbirths), comorbidity and sleep quality were assessed. Participants were considered to have a comorbidity if they were diagnosed with hypertension, heart failure, diabetes or hyperlipidaemia. The Pittsburgh Sleep Quality Index (PSQI) was used to assess sleep quality. Participants with a global PSQI score of > 7 were classified as having a sleep disorder [19]. Cronbach's alpha coefficient for PSQI in this study was 0.73.

#### 2.2.5. Demographic Variables

Age, education level (less than a bachelor's degree and bachelor's degree or above) and marital status (married, single/separated/divorced/widowed) were also included in the survey.

### 2.3. Data Analysis

Descriptive statistics were used to describe the sample characteristics. Latent class analysis (LCA) was conducted to identify the symptom patterns or clusters by jointly modelling 11 LUTS [20]. The fit indices are presented in Supporting Table 1. We began by specifying a 2-class model and then expanded it with additional classes until the model achieved the best fit and arrived at clinically meaningful classes. Although the Akaike and Bayesian information criteria did not reach a minimum for the seven fitted models, the 5-class model showed a large drop. Compared with the 5-class model, the 4-class model exhibited slightly better entropy and a higher probability of group membership. They were similar regarding other fit indices. Therefore, the 4-class model was determined to have a good overall fit and was the most parsimonious and readily interpretable model.

Associations between work-related factors or other covariates and latent clusters were examined using analysis of variance (ANOVA) and chi-square tests, as applicable. Subsequently, these factors were incorporated into a multivariate model using multinomial logistic regression analysis to explore the distinct risk factor profiles within each cluster. The ‘years in practice' variable was omitted from the logistic regression model because of its multicollinearity with age (correlation coefficient = 0.85). LCA was performed using Mplus Version 7 (Muthén and Muthén, College Station, TX, USA), and all other analyses were performed with Stata Version 14 (StataCorp LP, College Station, TX). Statistical significance was set at *p* < 0.05.

## 3. Results

### 3.1. Participants' Characteristics

The female nurses in the sample experienced on average 2.2 LUTS (Table 1). The most common symptom was urgency (51.03%), followed by SUI (34.98%). Nocturnal enuresis (2.61%) and frequency (1.28%) were the least common symptoms (Supporting Table 2). The nurses were relatively young with a mean age of 33.6 ± 7.5 years old (range 19–60 years). Most were married (81%), had obtained a bachelor's degree or higher (82%) and had given birth to at least one baby (76%). The prevalence rates of obesity and comorbidities were 6% and 4%, respectively. In contrast, about one-third reported poor sleep (33%), 70% drank fluid ≤ 1500 mL/day, and roughly 70% had the habit of delaying voiding. Regarding the work-related factors, 65% worked in Level A tertiary hospitals with a high workload, 62% worked night shifts, and nearly half (49%) worked more than 40 h/week. The average level of perceived stress was 6.6 ± 2.9.

### 3.2. Four Clusters of LUTS

**Figure 1:**
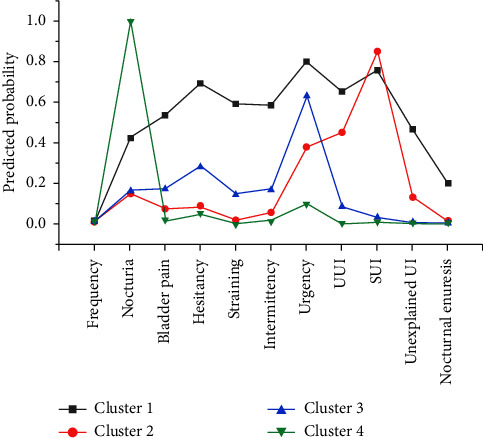
Item response probabilities within the four-cluster model. Abbreviations: SUI = stress urinary incontinence, UUI = urge urinary incontinence, UI = urinary incontinence.

**Figure 2:**
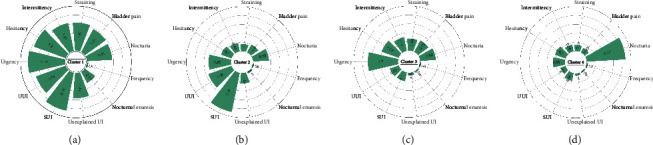
Average symptom severity score by cluster. *Note*: the results show the average score of symptom severity for Cluster 1 (a), Cluster 2 (b), Cluster 3 (c) and Cluster 4 (d). Abbreviations: SUI = stress urinary incontinence, UUI = urge urinary incontinence, UI = urinary incontinence.

LCA identified four clusters underlying the 11 symptoms, which were labelled according to their dominant symptoms (Figures 1 and 2). Participants in Cluster 1 (the Multiple Severe Symptoms cluster) had the most severe symptoms and accounted for 8% of female nurses with LUTS. This cluster consisted of nurses who reported six or more symptoms with a mean severity score greater than 9.72, which was the highest among the four clusters. Among the most common 9 symptoms, more than 40% experienced all symptoms. More than 60% of the participants experienced urgency, hesitancy, SUI and UUI.

Participants in Cluster 2 (the Incontinence Symptoms cluster) experienced several types of UI along with urgency and reported an average 2.3 symptoms. The dominant symptoms were SUI (86%) and UUI (44%). SUI was more severe, with a mean score of 2.24. This cluster was the second-largest symptom group, accounting for 31% of female nurses with LUTS.

Participants in Cluster 3 (the Urgency–Hesitancy Symptoms cluster) reported an average 1.7 symptoms. The dominant symptoms of this cluster were mild urinary urgency (63%) and hesitancy (28%), as indicated by a mean score of less than 2.0. Cluster 3 was the largest symptom cluster, accounting for 50% of female nurses with LUTS.

Participants in Cluster 4 (the Nocturia Symptom cluster) reported relatively few symptoms, accounting for 11% of female nurses with LUTS. Nocturia was the dominant symptom in this cluster, with few reports of the remaining 10 symptoms. Female nurses reported an average of 2.18 nocturia episodes per night.

### 3.3. Distinct Profiles of Female Nurses in the Four Clusters

**Figure 3:**
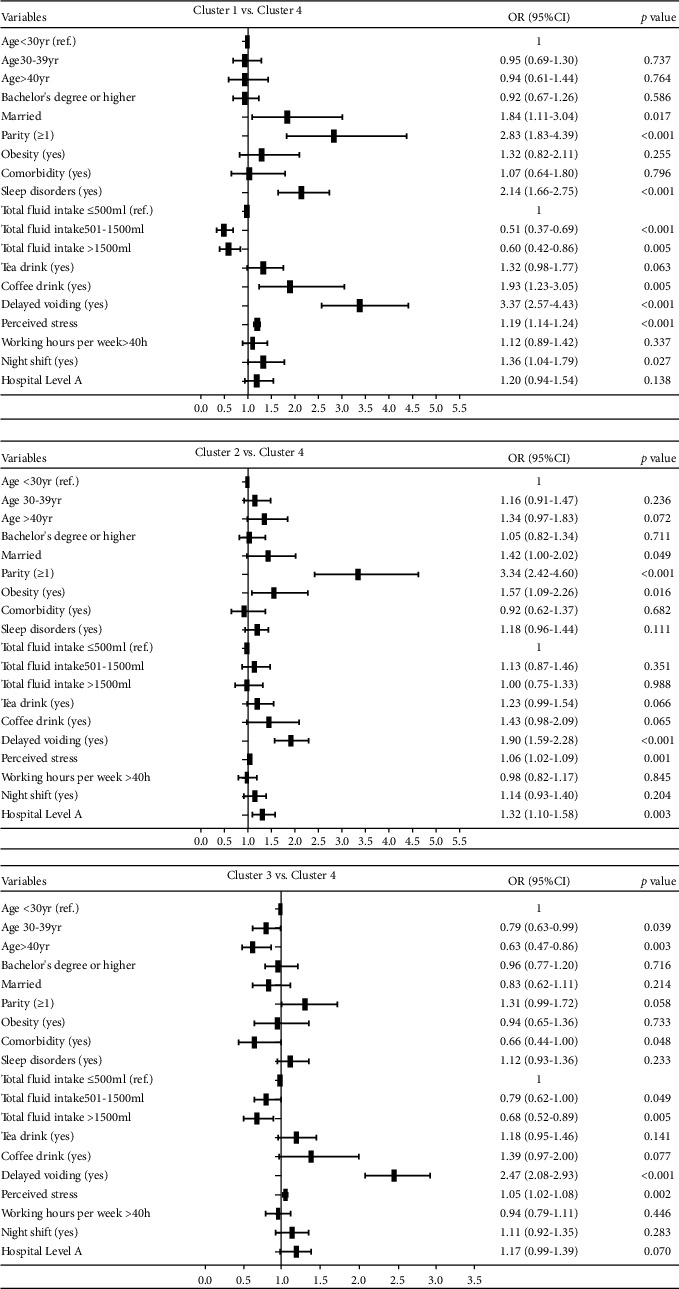
Multinomial logistic regression analysis showing the association of (1) demographics; (2) health conditions; (3) lifestyle factors; (4) work-related factors for Clusters 1 to 3, compared to Cluster 4.

The relationships between LUTS clusters and nurses' demographic characteristics, health-related factors, lifestyles and work-related factors are shown in Table 1 and Figure 3. Female nurses in Cluster 1 (the Multiple Severe Symptoms cluster) had the highest prevalence of comorbidities and poor sleep, and the highest proportions of drinking coffee, fluid intake ≤ 500 mL/day, delayed voiding, working more than 40 h/week and the highest perceived stress (Table 1). Compared to those in the minimal symptom group (Cluster 4), those in Cluster 1 were more likely to be married, have given birth to one or more children, have poor sleep, drink coffee, delay voiding, work night shifts, report higher perceived stress and report lower fluid drink (≤ 500 mL/day) (Figure 3).

Female nurses in Cluster 2 (the Incontinence Symptoms cluster) demonstrated the most advanced age and the highest prevalence of obesity, as well as the highest proportion of being married, giving birth to one or more children, having a bachelor's degree or above, working > 10 years and working in Level A hospitals (Table 1). Compared to those in the minimal symptom group (Cluster 4), female nurses in Cluster 2 were more likely to be obese, married, give birth to one or more children, delay voiding, work in Level A hospitals and perceive a higher level of stress (Figure 3).

Female nurses in Cluster 3 (the Urgency–Hesitancy Symptoms cluster) were the youngest and had the lowest prevalence of obesity and comorbidities, the lowest proportion of being married, having given birth to one or more children, having a bachelor's degree or above, working > 10 years and the second highest proportion of delayed voiding and perceived stress (Table 1). Compared to those in the minimal symptom group (Cluster 4), female nurses in Cluster 3 were more likely to be younger, drink less fluid (< 500 mL/day), delay voiding, perceive higher stress and less likely to report any comorbidities (Figure 3).

Female nurses in Cluster 4 (the Nocturia Symptom cluster) reported the lowest prevalence of poor sleep, the highest proportion of fluid intake > 1500 mL/day, the lowest proportions of coffee drinking, delayed voiding, working > 40 h/week, working in Level A hospitals and perceived the lowest level of stress (Table 1).

## 4. Discussion

Our study provides compelling insights into the LUTS profiles and contributing factors in a large sample of female nurses. Approximately 8% of symptomatic female nurses belonged to the Multiple Severe Symptoms cluster (Cluster 1) and reported to experience the most extensive range of symptoms as well as with the highest degree of symptom severity. This cluster has been previously identified in the BACH, LURN and EPIC studies, indicating a mixed pattern of storage and voiding symptoms [7]. Despite the symptom severity, this particular group displayed relatively low prevalence for the common risk factors. They were relatively younger (82% under 40 years old), healthier (only 6% with comorbidities), had fewer childbirths and had a lower BMI (8% obesity). Instead, our study highlighted the pronounced prevalence of work-related factors contributing to LUTS in this population. More than half of the nurses worked more than 40 h per week, 65% worked night shifts, 40% worked for more than 10 years, and 65% worked in Level A tertiary hospitals characterised by high workload demands. Female nurses in the identified cluster reported the highest levels of perceived stress, fluid restriction and delayed voiding. Our findings substantiate the vulnerability of the female nurses to LUTS, which appears to be primarily attributable to their demanding work environments. Furthermore, the findings underscore the importance of recognising LUTS among female nurses as an occupational hazard and that this issue remains an understudied workplace issue that warrants attention and tailored intervention strategies.

Nearly one-third of female nurses belonged to the cluster characterised by incontinence symptoms (Cluster 2), primarily SUI (86%) and UUI (44%). UI patterns have been previously reported to coexist with frequency and voiding symptoms [8]. The female nurses in Cluster 2 exhibited several distinct characteristics. They were typically the oldest of the participating nurses, were more likely to be married, had given birth to children and had the highest prevalence of obesity. These characteristics align with prior research, which has reported that age, marital status, parity and obesity were risk factors for UI [21], which are often associated with changes in pelvic floor muscles and hormones [8]. Furthermore, female nurses in this cluster demonstrated the highest proportion of working for over a decade in Level A hospitals with high workload demands. This finding suggests that the duration of exposure to a demanding work environment may contribute to developing of incontinence symptoms or exacerbating existing symptoms. Our study highlights the need for further research to investigate the possibility of a dose-related effect in the female nursing population, regarding the cumulative impact of occupational demands on incontinence symptoms.

The Urgency–Hesitancy Symptoms cluster (Cluster 3) identified in our female nurse sample was different from prior studies such as BACH, LURN and EPIC, which typically involve community-dwelling or care-seeking women. This particular cluster was the largest in our study, comprising approximately half of the nurses surveyed. Among the four identified clusters, female nurses in this cluster were the youngest (40% under age 30) and the least likely to be married, have children, have obesity or be diagnosed with chronic conditions. Given that the cluster was relatively young, approximately 30% had worked for 5 years or less. Conversely, they were mostly likely to work night shifts (67%). Distinctive behavioural patterns were also observed within this cluster, including a tendency to delay voiding and limit their fluids intake, with nearly 75% of participants reporting that they sometimes/often/always delayed voiding. These practices were not limited to Cluster 3 but were also prevalent among those in Cluster 1.

Furthermore, the entire sample reported infrequent urination during the day, with a very low prevalence of urinary frequency (1.28%). In contrast, nearly 51% of the participants reported experiencing urgency. This finding corresponds with previous research, showing that nearly half of nurses actively suppressed the urge to void during work hours [22]. This behaviour, often referred to as ‘infrequent voiding syndrome' or ‘nurses' bladder', may stem from the demanding nature of nursing roles, insufficient staffing and a lack of adequate break times [11, 13]. Nurses may choose to ignore their urge to void, postpone necessary restroom breaks or rush the voiding process to meet their job requirements [11]. Previous studies have indicated that chronically suppressing urges or delaying voiding that leads to an overfilled bladder can cause bladder hypertension, affect bladder perfusion and make it difficult to relax the sphincter [23, 24]. Ultimately, it affects both structure and function of the lower urinary tract [25]. Re-evaluating the culture of prioritising the care of others over oneself to achieve a better balance is necessary to address this issue. Furthermore, it is imperative to develop strategies that empower nurses to effectively manage both their personal health needs and overall well-being. This holistic approach to nursing practice and workplace culture may lead to more sustainable and effective healthcare delivery [26].

Female nurses in the minimal symptom cluster (Cluster 4) stood out for the lack of LUTS, except for nocturia. Nocturia clusters have also been observed in the BACH and EPIC studies [8, 9]. All the nurses in this cluster reported the need to wake up and urinate at least twice nightly. Compared with the other clusters, Cluster 4 had a low-risk profile. They reported the lowest perceived stress, the lowest proportion of individuals working more than 40 h per week and the lowest employment rate in high-workload hospitals. Moreover, they exhibited healthy behaviours, including the highest proportion of individuals drinking more than 1500 mL/day and the lowest prevalence of delayed voiding. Adequate fluid intake has been recognised as a protective factor against LUTS [27]. However, the prevalence of nocturia within this cluster raises questions regarding the potential underlying causes. Further research is warranted to investigate the factors contributing to nocturia.

This study has two strengths. First, using a representative sample of female nurses, we thoroughly examined the clinical spectrum of LUTS in this population. This allowed us to elucidate the distinct risk factor profiles associated with each of the four identified symptom clusters, thereby enriching global evidence regarding the prevalence and characteristics of LUTS among female nurses. The robustness of our sampling methodology enhances the generalisability of our findings to a broader nursing population. Second, our study identified unique profiles associated with each cluster, with a particular emphasis on work-related factors. This novel insight into the occupational dimensions associated with LUTS among nurses provides a nuanced understanding of the aetiology of this condition in high-stress healthcare environments. These findings have significant potential to inform existing interventions aimed at mitigating the burden of LUTS for nurses, potentially leading to improved occupational health strategies and policies in healthcare settings.

However, this study also has several limitations. First, our analysis was constrained by the symptoms included on the ICIQ-FLUTS. The absence of additional symptoms, such as postvoiding symptoms, may have affected the cluster results identified in our current analyses. Second, our cluster results are based on cross-sectional data from the baseline NURS dataset. A follow-up survey is currently underway to validate the robustness of these clusters. Replicating these findings with longitudinal data will offer valuable insights into whether these clusters truly represent distinct groups of female nurses with LUTS. Third, a comprehensive understanding of LUTS in female nurses requires further investigation into the underlying pathophysiology of these symptoms. This investigation should also encompass an evaluation of the effects of clinical treatment and management strategies based on cluster findings, particularly for subgroups afflicted with multiple severe symptoms.

## 5. Conclusions

This study identified four distinct LUTS clusters among female nurses, corroborating the common co-occurrence of urological symptoms. Our findings expand the current understanding of the clinical spectrum of LUTS by identifying the unique profiles associated with each symptom pattern. Work-related factors, including demanding workloads, elevated perceived stress, extended work hours, working night shifts, infrequent and delayed voiding behaviours and restricted fluid intake, significantly contributed to nurses' LUTS. Addressing these challenges necessitates not only increased staffing, but also a fundamental shift in workplace culture that prioritises nurses' well-being. Cultivating a culture of self-care, with a particular emphasis on bladder health promotion, is crucial for nursing professionals and, by extension, other employed women. These findings underscore the importance of implementing targeted interventions and policy changes to mitigate occupational risk factors associated with LUTS in healthcare settings.

## 6. Implications for Nursing Management

This study revealed distinctive symptom patterns among female nurses with LUTS, highlighting the specific work-related factors and behaviours associated with each pattern. Factors such as demanding workloads, high perceived stress and extended work hours, along with urinary behaviours such as delayed voiding and fluid restriction, were identified as significant contributors to LUTS in this sample of nurses. These results offer insights for developing customised intervention strategies and workplace initiatives aimed at enhancing nurses' well-being and promoting bladder health. Addressing these challenges requires not only increased staffing levels, but also a fundamental shift in workplace culture. Creating a supportive work environment and promoting nurses' self-care are imperative for enhancing nurses' well-being and ensuring the delivery of high-quality patient care.

## Figures and Tables

**Table 1:** Characteristics of participants according to the lower urinary tract symptom clusters.

**Characteristics**	**Total (*N* = 6735)**	**Cluster 1 (*N* = 546)**	**Cluster 2 (*N* = 2089)**	**Cluster 3 (*N* = 3357)**	**Cluster 4 (*N* = 743)**	**χ** ^2^ **/*F***	**p**
No. of symptoms, M (SD)	2.2 (1.6)	6.0 (1.5)	2.3 (1.6)	1.7 (1.0)	1.1 (0.3)	1400.0	<0.001

*Demographics*							
Age, M (SD)	33.6 (7.5)	34.1 (6.9)	35.9 (7.5)	31.9 (7.0)	34.1 (8.4)	48.1	<0.001
<30 years	2075 (30.8)	130 (23.8)	363 (17.4)	1352 (40.3)	230 (31.0)	388.2	<0.001
30–39 years	3368 (50.0)	317 (58.1)	1158 (55.4)	1547 (46.9)	346 (46.6)		
40–60 years	1292 (19.2)	99 (18.1)	568 (27.2)	458 (13.6)	167 (22.5)		
Marital status						395.6	<0.001
Single, separated or divorced	1275 (18.9)	46 (8.4)	146 (7.0)	921 (27.4)	162 (21.8)		
Married	5460 (81.1)	500 (91.2)	1943 (93.0)	2436 (72.6)	581 (78.2)		
Education						49.2	<0.001
Below a bachelor's degree	1229 (18.3)	94 (17.2)	286 (13.7)	712 (21.2)	137 (18.4)		
Bachelor's degree or higher	5506 (81.7)	452 (82.8)	1803 (86.3)	2645 (78.8)	606 (81.6)		

*Health condition*							
^#^Parity						488.1	<0.001
0	1637 (24.4)	73 (13.4)	194 (9.3)	1149 (34.5)	221 (29.9)		
≥1	5063 (75.6)	471 (86.6)	1890 (90.7)	2183 (65.5)	519 (70.1)		
^#^Obesity						35.3	<0.001
No	6303 (93.7)	504 (92.5)	1905 (91.3)	3192 (95.2)	702 (94.6)		
Yes	425 (6.3)	41 (7.5)	182 (8.7)	162 (4.8)	40 (5.4)		
Comorbidity						46.2	<0.001
No	6442 (95.7)	511 (93.6)	1960 (93.8)	3267 (97.3)	704 (94.8)		
Yes	293 (4.3)	35 (6.4)	129 (6.2)	90 (2.7)	39 (5.2)		
^#^Sleep disorder						119.1	<0.001
No	4459 (66.7)	251 (46.7)	1411 (67.9)	2513 (67.5)	546 (74.4)		
Yes	2226 (33.3)	287 (53.3)	668 (32.1)	1083 (32.5)	188 (25.6)		

*Lifestyle factors*							
Fluid intake (ml/day)						95.2	<0.001
≤500	1202 (17.9)	143 (26.2)	275 (13.2)	676 (20.1)	108 (14.5)		
501–1500	3816 (56.7)	260 (47.6)	1216 (58.2)	1925 (57.3)	415 (55.9)		
>1500	1717 (25.5)	143 (26.2)	598 (28.6)	756 (22.5)	220 (29.6)		
Tea drink						39.7	<0.001
No	5135 (76.2)	404 (74.0)	1500 (71.8)	2654 (79.1)	577 (77.7)		
Yes	1600 (23.8)	142 (26.0)	589 (28.2)	703 (20.9)	166 (22.3)		
Coffee drink						11.6	0.009
No	6228 (92.5)	489 (89.6)	1930 (92.4)	3106 (92.5)	703 (94.6)		
Yes	507 (7.5)	57 (10.4)	159 (7.6)	251 (7.5)	40 (5.4)		
Delayed voiding						209.0	<0.001
Never/rarely	2024 (30.1)	101 (18.5)	705 (33.8)	854 (25.4)	364 (49.0)		
Sometimes/often/always	4711 (69.9)	445 (81.5)	1384 (66.2)	2503 (74.6)	379 (51.0)		

*Work-related factors*							
Perceived stress	6.6 (2.9)	7.8 (2.5)	6.4 (3.0)	6.6 (2.8)	5.9 (2.9)	25.2	<0.001
Working hours per week						17.8	<0.001
≤40 h	3433 (51.0)	241 (44.1)	1111 (53.2)	1681 (50.1)	400 (53.8)		
>40 h	3302 (49.0)	305 (55.9)	978 (46.8)	1676 (49.9)	343 (46.2)		
Night shift						87.4	<0.001
No	2530 (37.6)	189 (34.6)	935 (44.8)	1097 (32.7)	309 (41.6)		
Yes	4205 (62.4)	357 (65.4)	1154 (55.2)	2260 (67.3)	434 (58.4)		
Working years						433.4	<0.001
≤5 years	1476 (21.9)	84 (15.4)	197 (9.4)	1020 (30.4)	175 (23.6)		
5–10 years	2500 (37.1)	243 (44.5)	749 (35.9)	1243 (37.0)	265 (35.7)		
>10 years	2759 (41.0)	219 (40.1)	1143 (54.7)	1094 (32.6)	303 (40.8)		
Hospital level						10.7	0.014
Level B tertiary hospital	2361 (35.1)	193 (35.4)	682 (32.7)	1197 (35.7)	289 (38.9)		
Level A tertiary hospital	4374 (64.9)	353 (64.6)	1407 (67.3)	2160 (64.3)	454 (61.0)		

Abbreviation: SD, standard deviation.

^#^missing data exists.

## Data Availability

The data that support the findings of this study are available from the corresponding author upon reasonable request.

## Supporting Information

Supporting Table 1: Model fit indices derived from the latent class analysis on models with two to seven classes. Supporting Table 2: Prevalence of lower urinary tract symptoms in symptomatic nurses.
