# Supplementary material for: Work-Related Stress and Behavioural Correlates of Lower Urinary Tract Symptom Profiles in Female Nurses: A Latent Class Analysis Based on the Nurse Urinary Related Health Study: Four Profiles of Lower Urinary Tract Symptoms in Female Nurses
Source: J Nurs Manag. 2024 Oct 17;2024:7318901. doi: 10.1155/2024/7318901 (PMC11919071; doi:10.1155/2024/7318901)
Supplement: Supporting Information — Additional supporting information can be found online in the Supporting Information section. [file 7318901.f1.docx]

Supplementary Table .

Table 1.Model fit indices derived from the latent class analysis on models with two to seven classes

| Model | K | Log(L) | AIC | BIC | SABIC | Entropy | VLMR | BLMt *p*-value |
| --- | --- | --- | --- | --- | --- | --- | --- | --- |
| 2 | 23 | -30803.04 | 61652.08 | 61808.83 | 61735.74 | 0.81 | 0.012 | <0.001 |
| 3 | 35 | -29989.30 | 60048.59 | 60287.12 | 60175.90 | 0.78 | <0.001 | <0.001 |
| 4 | 47 | -29595.93 | 59285.85 | 59606.16 | 59456.81 | 0.78 | <0.001 | <0.001 |
| 5 | 59 | -29085.94 | 58289.88 | 58691.97 | 58504.48 | 0.78 | <0.001 | <0.001 |
| 6 | 71 | -28971.39 | 58084.78 | 58568.65 | 58343.03 | 0.76 | <0.001 | <0.001 |
| 7 | 83 | -28876.06 | 57918.11 | 58483.76 | 58220.01 | 0.78 | <0.001 | <0.001 |

Table 2. Prevalence of lower urinary tract symptoms in symptomatic nurses.

| Types of LUTS | Nurses (*N* = 6735) |
| --- | --- |
| Urinary urgency, *n* (%) | 3437 (51.03) |
| Stress urinary incontinence, *n* (%) | 2356 (34.98) |
| Urgency urinary incontinence, *n* (%) | 1603 (23.80) |
| Unexplained urinary incontinence, *n* (%) | 571 (8.48) |
| Nocturia, *n* (%) | 1833 (27.22) |
| Bladder pain, *n* (%) | 1056 (15.68) |
| Hesitancy, *n* (%) | 1563 (23.21) |
| Intermittency, *n* (%) | 1032 (15.32) |
| Straining, *n* (%) | 883 (13.11) |
| Frequency, *n* (%) | 86 (1.28) |
| Nocturnal enuresis, *n* (%) | 176 (2.61) |
